# Fantastic Lists and Where to Find Them: Implementation of Centralised Jobs Lists Into Psychiatric Workplaces

**DOI:** 10.1192/bjo.2024.428

**Published:** 2024-08-01

**Authors:** Samriddhi Sain, Christos Charalambous

**Affiliations:** Oxleas NHS Foundation Trust, London, United Kingdom

## Abstract

**Aims:**

This quality improvement project aims to address the current gaps in safe handover between doctors on psychiatric wards by implementing a “live jobs list” that can be remotely accessed and edited by all members of the ward medical team. It should create accountability between different members of the medical team and allow colleagues to track which jobs have been started, completed or are not yet assigned; avoiding duplication or noncompletion of outstanding ward tasks.

**Methods:**

Qualitative surveys were sent out to junior doctors working within inpatient psychiatric wards. The survey focussed on identifying the opinions of doctors about jobs lists and their views regarding collaborative vs. individualised lists. The survey was sent out prior to creating ward-specific online channels with collaborative task lists that could be accessed by the whole team. The survey was then repeated after 4 months of this system being implemented to see how it had changed the opinions of the doctors using it.

**Results:**

Of 21 participants, 95.2% had used individualised jobs lists (IJLs) with 52.4% having negative experiences of these. Only 76.2% of participants had used centralised jobs lists (CJLs) and 42.9% had negative experiences with these. Overall, 61.9% of participants preferred CJLs.

Negative experiences with IJLs focused on lack of accountability, duplication of tasks and unsafe handover. The negative experiences of CJLs revolved around colleagues not correctly using the platform and the process being time-consuming compared with IJLs due to preference of layout and user interface.

The MS Teams CJL was then implemented into multiple wards within an inpatient psychiatry setting. After 4 months of use, the majority of participants (80.9%) were in favour of CJLs; this could be categorised into three main reasons: 1) reduced risk of overlooking or duplicating tasks, 2) safer handover within the team especially due to shift patterns and sickness, 3) accountability within the wider team for clinical tasks. Those who preferred IJLs stated that the newer system was “difficult to adapt to” and that they lacked senior input on how to incorporate it.

**Conclusion:**

Amongst inpatient psychiatry doctors, the use of a CJL has shown to be preferable due to improvements in efficiency, safety and accountability. Although there are barriers to overcome, namely regarding the initial implementation of the system and lack of customisation to individual preferences, this can be explored in the future with the aim to further increase the appeal to doctors working within a ward team.

